# Lipid productivity in limnetic *Chlorella* is doubled by seawater added with anaerobically digested effluent from kitchen waste

**DOI:** 10.1186/s13068-018-1064-5

**Published:** 2018-03-14

**Authors:** Liqun Jiang, Lijie Zhang, Changliang Nie, Haiyan Pei

**Affiliations:** 1 School of Environmental Science and Engineering, Shandong University, No. 27 Shanda Nan Road, Jinan, 250100 China; 2 Shandong Provincial Engineering Centre on Environmental Science and Technology, No. 17923 Jingshi Road, Jinan, 250061 China

**Keywords:** Seawater, Anaerobically digested effluent from kitchen waste, Limnetic microalgae, Lipid

## Abstract

**Background:**

An economical strategy for producing microalgae as biofuel feedstock is driven by the freshwater and nutrients input. In this study, seawater was applied to limnetic algal cultivation and the behavior of algae in seawater media was observed including growth, lipid synthesis, and ultrastructure. To make seawater cater algae, a kind of wastewater, anaerobically digested effluent from kitchen waste (ADE-KW), was used as nutrient sources.

**Results:**

Pure seawater cannot support the growth demand of freshwater microalga, due to high salinity and lack of nutrients. However, it is the conditions triggered the algae to synthesize lipids of 60%, double of lipid content in standard medium BG11. Introducing 3 or 5% ADE-KW (volume percentage) into seawater made algal growth reach the level attained in BG11, while lipid content compared favourably with the level (60%) in pure seawater. This method achieved the goal of fast growth and lipid accumulation simultaneously with the highest lipid productivity (19 mg/L  day) at the exponential stage, while BG11 obtained 10.55 mg/L  day at the stationary stage as the highest lipid productivity, almost half of that in seawater media. Moreover, the condition for highest lipid productivity enlarged algal cells compared to BG11. Under the condition for highest lipid productivity, *Chlorella sorokiniana* SDEC-18 had enlarged cells and increased settling efficiency compared to BG11, which facilitated harvest in an energy saving way.

**Conclusions:**

The results suggested that combining seawater with ADE-KW to cultivate microalgae had a double function: nutrients and water for algal growth, and high salinity for stimulating lipid accumulation. If this technology was operated in practice, freshwater and non-waste nutrient consumption would be completely obviated.

**Electronic supplementary material:**

The online version of this article (10.1186/s13068-018-1064-5) contains supplementary material, which is available to authorized users.

## Background

Algal biofuels are seriously considered as replacement for the unsustainable fossil fuels due to their attractive characteristics including carbon-neutrality, high biomass productivity, and the associated high lipid yields [1, 2]. However, there is not yet consensus on economical and environmentally beneficial methods for large scale production of algal biofuels, because of realistic challenges like the competition for fresh water, nutrient deprivation, and energy consumption in harvesting [3]. In algae–biofuel production systems, water is mainly used for algae cultivation and process cooling. The usage of freshwater would be reduced considerably if seawater or saltwater played a role in the two operations. Fortunately, seawater, covering 71% of the earth’s surface, is a more abundant resource than freshwater, and salt aquifers are available in deserts where freshwater is scarce.

Currently, seawater is mainly used for diluting wastewater for marine algae cultivation, while, in fact, artificial seawater was the primary choice for trials to stimulate lipid accumulation and avoid bio-invasion from other freshwater microorganisms. Cai et al. [4] explored the potential use of artificial seawater to produce lipids in the marine microalgae *Synechocystis* sp. and *Nannochloropsis salina* with anaerobic digestion effluent as the nutrient source. The results suggested a feasible way to yield suitable fatty acids for biodiesel. A similar finding was obtained for *N. salina* in outdoor conditions with artificial seawater and anaerobic digestion effluent [5]. Zhao et al. [6] isolated a marine microalgae *Nannochloropsis gaditana* from a beach and cultivated it in f/2-Si medium enriched with natural seawater. The highest biomass (572 mg/L) and lipid content (18%) were achieved in seawater without freshwater. Regarding the invasion of algal competitors and predators, Bartley et al. [7] added ocean salt to culture medium to get an appropriate salinity for fast growth of marine algae and low density of invading organisms.

In respect of freshwater microalgae, an engineering strategy using deep-sea water as the medium was applied to promote growth and oil accumulation of the microalga *Chlorella sorokiniana* CY1 by Chen et al. [8]. The addition of 20% deep-sea water to the culture medium effectively enhanced the cell growth and oil accumulation of the algae. Jung et al. [9] found retardation in cell growth when more than 10% seawater was added to BG11 medium for cultivation of *Scenedesmus obliquus*, and higher fatty acid yields relative to those in the standard BG11. Some reports added NaCl to the medium to build a salt-stress environment for hyperaccumulation of lipids in limnetic algae, and the results were satisfactory [10]. In contrast to the case for marine algae, seawater used in production of freshwater algae acts as a supplement, not a replacement for the standard freshwater medium, due to its high salinity. However, a method to culture freshwater algae with seawater is more urgent than that for marine algae, because many more freshwater algae strains have been isolated and are available for producing biofuel feedstock [11].

Though seawater has great potential for cultivating limnetic microalgae, there are also several challenges on the way, including the low contents of nitrogen and phosphorous that retard algae growth. Nutrient supply is an important parameter. Nitrogen and phosphorus are the main requirements in algal cells (7% nitrogen and 1% phosphorus) [12]. This cost of nutrient supply can be managed by introducing wastewater streams in the algal cultivation system, like some researchers have done [13, 14]. Besides the main nutrients, trace elements play an important role in rich algal growth, and are also costly. Fortunately, seawater contains abundant mineral elements supporting marine biology [9], which might also be used for freshwater algal activity. Therefore, cultivating freshwater microalgae with seawater has practical value and a good prospect for application.

Following these concerns and hypotheses, we chose anaerobically digested effluent from kitchen waste (ADE-KW) as the nutrient source, as previously reported [15]. Kitchen waste occupies a large part of daily municipal waste and anaerobic digestion technology is an effective and environment-friendly way to deal with it. At the end of this process, ADE-KW is produced in large amounts and contains abundant nutrients accessible to microalgae. Moreover, infrastructure employed by anaerobic digestion process of kitchen waste can be used for algae-based biodiesel production.

Focusing on the cost of freshwater and nutrient chemicals and energy of harvest in algae production process, the main aims in our study included: (1) understanding the behavior of limnetic microalgae in a medium consisting of 100% seawater, (2) exploring the feasibility of using ADE-KW as nutrient source for algae cultured in seawater, (3) establishing a cultivation system in the absence of freshwater, and (4) find a simple and economical way to stimulate lipid accumulation in freshwater microalgae.

## Methods

### Algal species

The raw material, microalgae *C. sorokiniana* SDEC-18 (GenBank Accession No.: KY355143), was obtained from the local freshwater lake (Quancheng Lake in Jinan) that had been slightly polluted by wastewater. The isolation and identification procedures were done as in the previous report [16].

The isolated microalgae were preserved in BG11 medium which contains: 1.5 g/L NaNO_3_, 40 mg/L K_2_HPO_4_, 75 mg/L MgSO_4_·7H_2_O, 36 mg/L CaCl_2_·2H_2_O, 6 mg/L citric acid, 6 mg/L ferric ammonium citrate, 1 mg/L EDTA-Na_2_, 20 mg/L Na_2_CO_3_, and 1 mL/L A_5_. A_5_ is a trace metal solution containing 2.86 g/L H_3_BO_3_, 1.86 g/L MnCl_2_·4H_2_O, 0.22 g/L ZnSO_4_·7H_2_O, 0.39 g/L Na_2_MoO_4_·2H_2_O, 0.08 g/L CuSO_4_·5H_2_O, and 0.05 g/L Co(NO_3_)_2_·6H_2_O.

### Cultivation methods

The algae reaching the late exponential phase were recovered by centrifugation and washed three times, and then suspended in 15 mg/L NaHCO_3_ solution before inoculation. The microalgae were next transferred to a 1 L conical flask containing the seawater supplemented with anaerobically digested effluent from kitchen waste (ADE-KW) with an initial optical density of 0.2 at 680 nm, read with a UV–Vis spectrophotometer (UV-2450, Shimadzu, Japan). The seawater was collected from Qingdao and filtered through 0.22 μm membranes before use. The ADE-KW was provided by Shandong Shifang Environmental Protection & Bio-Energy Co. The collected ADE-KW was filtered through six layers of gauze to remove the insoluble solids. The characteristics of the seawater and wastewater were analyzed including turbidity (seawater 0.28 NTU, ADE-KW 1410 NTU), pH (seawater 8.23, ADE-KW 8.55), and other compositions shown in Table S1 (Additional file 1).

Five ADE-KW loadings (1, 3, 5, 8 and 15%) were applied to modify the seawater medium, and BG11 medium and pure seawater (0% ADE-KW) were also employed as controls.

The cultivation conditions were as follows: continuous illumination from fluorescent tubes, which provided about 80 μmol/m^2^ s light intensity as detected by an irradiance sensor (ZDS-10, Shanghai Cany Precision Instrument, China); temperature 25 ± 1 °C. All of the batch experiments were conducted in triplicate.

### Biomass accumulation

Microalgal growth was monitored every 24 h by measuring optical density at 680 nm (OD_680_). The dry cell mass (DCM) was calculated by the following equation:

1 $${\text{DCM}}\, = \,0. 6 5 7 {\text{OD}}_{ 6 80} - 0.0 8 4 1\;R^{ 2} \, = \,0. 9 9 2 6,$$

The biomass productivity (mg/L  day) was calculated according to equation:

2 $$P_{\text{b}}\, = \,{{\left( {X_{ 2} {-}X_{ 1} } \right)} \mathord{\left/ {\vphantom {{\left( {X_{ 2} {-}X_{ 1} } \right)} {\left( {T_{ 2} {-}T_{ 1} } \right)}}} \right. \kern-0pt} {\left( {T_{ 2} {-}T_{ 1} } \right)}},$$

where *X*_2_ and *X*_1_ are the dry cell mass (DCM) (mg/L) at time *T*_2_ and *T*_1_, respectively. The specific growth rate (*μ*) of microalgae in the logarithmic phase was calculated by the following equation [17]:

3 $$\mu\, = \,{{\left[ {{\text{Ln}}\left( {N_{ 2} } \right)\,{-}\,{\text{Ln}}\left( {N_{ 1} } \right)} \right]} \mathord{\left/ {\vphantom {{\left[ {{\text{Ln}}\left( {N_{ 2} } \right){-}{\text{Ln}}\left( {N_{ 1} } \right)} \right]} {\left( {T_{ 2} {-}T_{ 1} } \right)}}} \right. \kern-0pt} {\left( {T_{ 2} \,{-}\,T_{ 1} } \right)}},$$

where *N*_1_ and *N*_2_ present the dry cell mass (DCM) (mg/L) at time *T*_1_ and *T*_2,_ respectively.

### Cell morphology and size

Before harvesting algae, observations of algal cell size and morphology were performed using an inverted fluorescence microscope (Ti-s, Nikon, Japan), and then, statistical analysis of the distributions of cell diameters and the mean diameters of algal cell was computed with NIS-Elements D 4.20.00 software.

To directly assess the surface information and morphology of microalgae cultivated in BG11 medium and seawater, electron microscope (SU-70, Hitachi, Japan) and scanning electron microscopy (SEM) were used. To prepare algal sample for SEM, the algal cells were centrifuged at 4000 rpm for 5 min. The pellets were pre-fixed with 2.5% glutaraldehyde at 4 °C for 4 h, washed with phosphate buffer solution and post-fixed with 1% osmium-tetroxide for 1 h, and then washed again with phosphate buffer solution. After that, samples were consecutively treated with 50, 75, 90, and 100% ethanol solutions (15 min each) and dried with a vacuum drier. The completely dry samples were then mounted on a copper stub, coated with gold, and examined with a scanning electron microscope (SEM, Rili SU-70) at 5 kV.

### Photosynthetic pigments

Pigment content was determined according to Lichtenthaler [18]. In detail, 2 mL of culture was centrifuged at 10,000 rpm for 5 min, supernatant was discarded and the pellet was mixed with 99.9% methanol and incubated in the dark for 24 h at 45 °C. After incubation, pigment content was determined using the following formulae:

Chlorophyll a: Chl-a (mg/L) = 16.72 A_665.2_ − 9.16 A_652.4_
Chlorophyll b: Chl-b (mg/L) = 34.09 A_652.4_ − 15.28 A_665.2_

4 $${\text{Carotenoids}}\,=\,{{\left( {{1}000{\text{ A}}_{{{47}0}}\;{-}\;{1.63} \;{\text{Chl-a}}\;{-}\;{1}0{4.9}\;{\text{Chl-b}}} \right)} \mathord{\left/ {\vphantom {{\left( {{1}000{\text{ A}}_{{{47}0}} \;{-}\;{1.63}\;{\text{Chl-a}}\;{-}\;{1}0{4.9}{\text{Chl-b}}} \right)} {221}}} \right. \kern-0pt} {221}},$$

absorbencies at 470, 652.4, and 665.2 nm were corrected for turbidity by subtracting absorbance at 750 nm.

### Relative electrical conductivity

The relative electrical conductivity was measured according to previously reported methods of Kohno et al. [19]. Microalgae samples were collected of 20 mL volume and then centrifuged. After discarding supernatant, the microalgae pellets were washed three times with distilled water to wash out the nutrients adhering to cells, 10 mL distilled water was added for measuring the initial electrical conductivity *R*_0_ and an electrical conductivity *R*_1_ after standing for 30 min. Then that microalgal mixture was heated in a boiling water bath for 15 min and cooled to room temperature before again measuring the detecting the electrical conductivity (*R*_2_). The relative electrical conductivity (%) was calculated according to the equation:

5 $${\text{Relative electrical conductivity }}\left( \% \right)\,= \,{{\left( {R_{ 1} {-}R_{0} } \right)} \mathord{\left/ {\vphantom {{\left( {R_{ 1} {-}R_{0} } \right)} {\left( {R_{ 2} {-}R_{0} } \right)}}} \right. \kern-0pt} {\left( {R_{ 2} {-}R_{0} } \right)}}\; \times \; 100\% ,$$

### Lipid content and fatty acid profile

Every 2 days, 100 mL algae culture was harvested and dried for lipid analysis. The chloroform–methanol method was used to extract lipid from the dried algae powder, and then, the lipid was quantified gravimetrically following the description of Song et al. [20]. The average lipid productivity (*P*_L_, mg/L  day) was calculated by the following equation:

6 $$P_{\text{L}}\,= \,{{\left( {{\text{DCM}}\; \times \;{\text{LC}}} \right)} \mathord{\left/ {\vphantom {{\left( {{\text{DCM}}\; \times \;{\text{LC}}} \right)} {\left( {T\; \times \; 100} \right)}}} \right. \kern-0pt} {\left( {T\; \times \; 100} \right)}},$$

where DCM and LC represent the final biomass mass (mg/L) and the total lipid content (%), respectively, and *T* was the cultivation period (days).

Fatty acids were extracted from the dried algal powder using the two-step in situ methyl esterification process, and then examined through the GC–MS method following the procedure of Ji et al. [21].

### Fluorescence images and confocal microscopy image

Microalgae cells in the logarithmic and stationary stage were harvested by centrifuging at 8000 rpm for 5 min and pellets were washed 2–3 times with phosphate buffered saline (PBS) and resuspended in PBS solution to an optical density of 0.8 at 680 nm. After that, the resuspended algal cells were re-concentrated tenfold to ensure that there were 30–40 cells in the field of view. Next, 750 μL of the concentrated algal solution was placed in a centrifuge tube and 250 μL of 25% dimethyl sulfoxide (DMSO) was added [22]. Nile Red dye was added to each tube to make its concentration 1.5 μg/mL. After that, the tubes were shaken for 1 min and incubated in the dark for 10–15 min.

After staining, the distribution of intracellular neutral lipid (NL) in microalgal cells was studied on an inverted fluorescence microscope (Ti-s, Nikon, Japan) equipped with NIS-Elements D 4.20.00 software. The excitation wavelength was set to blue light and the emission wavelength at 585 ± 40 nm. The three types of images (bright-field, chlorophyll autofluorescence, and merged images) were used to study cellular morphology as well as the accumulation of NL droplets. The calculation of fluorescence intensity in microalgae cells was implemented using ImageJ software.

### Transmission electron microscopy

Algae taken from the logarithmic and stationary phase were made into block masses, which were chipped into tissue blocks of 1 mm^3^ on ice and promptly put into 3% glutaraldehyde in a cacodylate buffer at 4 °C for 2 h and subsequently post-fixed in a 1% osmium-tetroxide phosphate buffer for 1 h. Next, the samples were dehydrated in a graded ethanol series with acetone, and permeated and embedded in epoxide resin. Ultrathin sections were prepared, stained with uranyl acetate and lead citrate, and examined using a JEOL-1200EX transmission electron microscope (Hitachi Electronic Instruments, Tokyo, Japan).

### Statistical analysis

Results are presented in the form of mean value ± standard deviation from three independent experiments. The differences between experimental groups were analyzed using one-way analysis of variance (ANOVA). A difference was considered statistically significant when *p* < 0.05.

## Results and discussion

### Algal viability in seawater with nutrients supplemented from ADE-KW

Strains of the green algae *Chlorella* are the most promising microalgae for biodiesel applications, as it is common in freshwater and known to have high adaptability to environmental variation [23]. BG11 is the most commonly used freshwater medium for its cultivation, while seawater has many advantages, including being rich in the inorganic minerals needed by microalgae [8], and more abundant than freshwater.

**Fig. 1:**
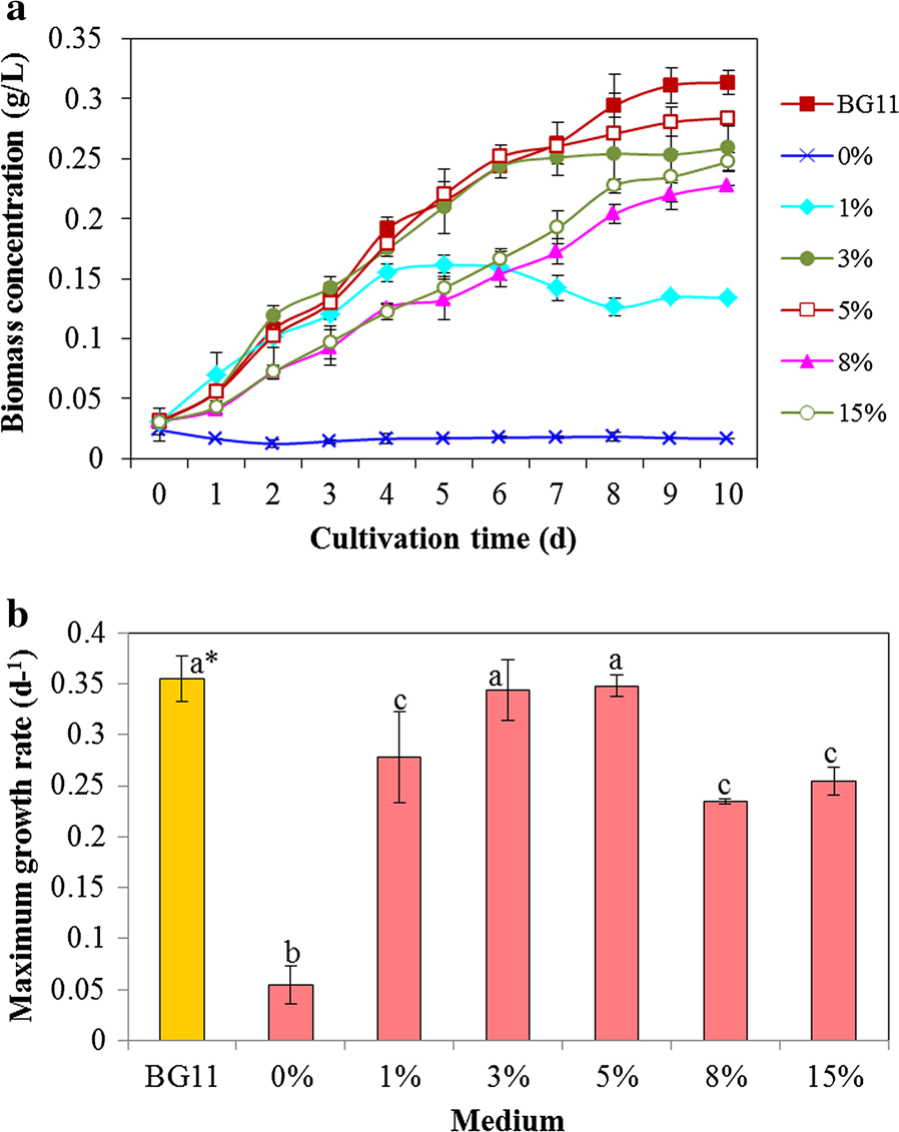
Biomass concentration (**a**) and maximum growth rate (**b**) of *Chlorella sorokiniana* SDEC-18 grown in BG11 and seawater supplemented with different volume percentages (0, 1, 3, 5, 8, and 15%) of anaerobically digested effluent from kitchen waste. *Data followed by different letters are significantly different by Duncan’s test at *p* < 0.05

In this study, the potential of seawater as a complete substitute for BG11 medium was examined by *Chlorella* cultivation method. The measurement of ion concentrations showed that many essential elements in BG11 for algal growth can be provided by seawater, like Na^+^, K^+^, Ca^2+^, Mg^2+^, Fe^3+^, Cu^2+^, and Zn^2+^ (Additional file 1: Table S1). However, the direct use of seawater for cultivation of *C. sorokiniana* SDEC-18 was not feasible, due to its very high salt concentration and lack of nutrients, such as N and P. The little growth of SDEC-18 in pure seawater (the line indicated by 0% in Fig. 1a) demonstrated this application infeasible.

The wastewater with sufficient nitrogen and phosphorus inspired a unique way to solve the application of seawater in algae cultivation. Five loadings of ADE-KW (1, 3, 5, 8, and 15%) were applied to modify the seawater medium. Algal biomass was promoted by the addition of ADE-KW at all loadings, compared to almost no growth in pure seawater (0%) (Fig. 1a). Especially in 3 and 5% media, algae grew ideally, producing as much biomass as in the standard BG11 within the first 6 days, and then, slow growth was exhibited after 6 days due to consumption of nutrients. The maximum growth rate at the exponential phase for algae in 3 and 5% media also exhibited no significant difference from that in BG11, as shown in Fig. 1b (*p* < 0.05). Although *C. sorokiniana* SDEC-18 in 1% ADE had a high maximum growth rate, it had unsatisfactory biomass accumulation due to the short exponential period. These results indicated that deficiency in nutrients was a primary reason for lack of growth of algae in seawater. Compared to algae in 3 and 5% media, retardation in cell growth and lower maximum specific growth rate were observed in 8%, and 15% ADE-KW in the initial days, which might have resulted from high turbidity (115 ± 2 NTU in 8% ADE-KW, 217 ± 5 NTU in 15% ADE-KW) and/or high ammonia levels (177.29 ± 14.07 mg/L in 8% ADE-KW, 333.61 ± 4.75 mg/L in 15% ADE-KW).

In summary, seawater supplemented with 3 and 5% ADE-KW was relatively desirable for SDEC-18 cultivation, and the dry mass (0.259 and 0.284 g/L) and cell numbers (6.83 × 10^8^ and 7.17 × 10^8^ cells/L) on the final day as well as the biomass productivity (22.8 and 25.3 mg/L  day) are presented in Table 1.

**Table 1:** Final biomass concentration, cell density and cell mass, and biomass productivity of *Chlorella sorokiniana* SDEC-18 grown in BG11 and in seawater supplemented with different volume percentages (1, 3, 5, 8, and 15%) of anaerobically digested effluent from kitchen waste

Medium	Biomass concentration (g/L)	Cell density (× 10^8^/L)	Biomass productivity (mg/L day)	Cell mass (ng/cell)
BG11	0.313 ± 0.010^a^*	15.75 ± 0.07^a^	28.3 ± 0.07^a^	0.20 ± 0.002^a^
0%	0.017 ± 0.000^b^	1.67 ± 0.28^b^	0 ± 0.929^b^	0.10 ± 0.03^a^
1%	0.134 ± 0.001^c^	2.75 ± 0.07^bc^	10.3 ± 0.976^c^	0.49 ± 0.02^bcd^
3%	0.259 ± 0.019^d^	6.83 ± 0.28^de^	22.8 ± 1.254^d^	0.38 ± 0.00^be^
5%	0.284 ± 0.003^e^	7.17 ± 0.42^e^	25.3 ± 0.047^e^	0.40 ± 0.02^bce^
8%	0.228 ± 0.000^f^	5.25 ± 1.06^df^	19.7 ± 0.047^f^	0.43 ± 0.06^bcde^
15%	0.247 ± 0.008^d^	7.0 ± 0.14^e^	21.6 ± 0.418^d^	0.35 ± 0.01^e^

* Data in the same column followed by different letters are significantly different by Duncan’s test at *p* < 0.05

Ability of *C. sorokiniana* SDEC-18 to grow in seawater reduces the freshwater demand. Anaerobically digested effluent from kitchen waste provides luxury nutrients and a hope to make this ability meaningful. Moreover, when using *Chlorella* strains to treat ADE-KW, dilution always comes before the treatment [14], which might be a waste of freshwater. Now, seawater addresses this urgent challenge.

### Lipid accumulation in algae cultured with seawater combined with ADE-KW

#### Lipid content

For presenting lipid accumulation of *C. sorokiniana* SDEC-18, data and image were collected through total lipid extraction, Nile Red staining, and ultrastructure observation.

**Fig. 2:**
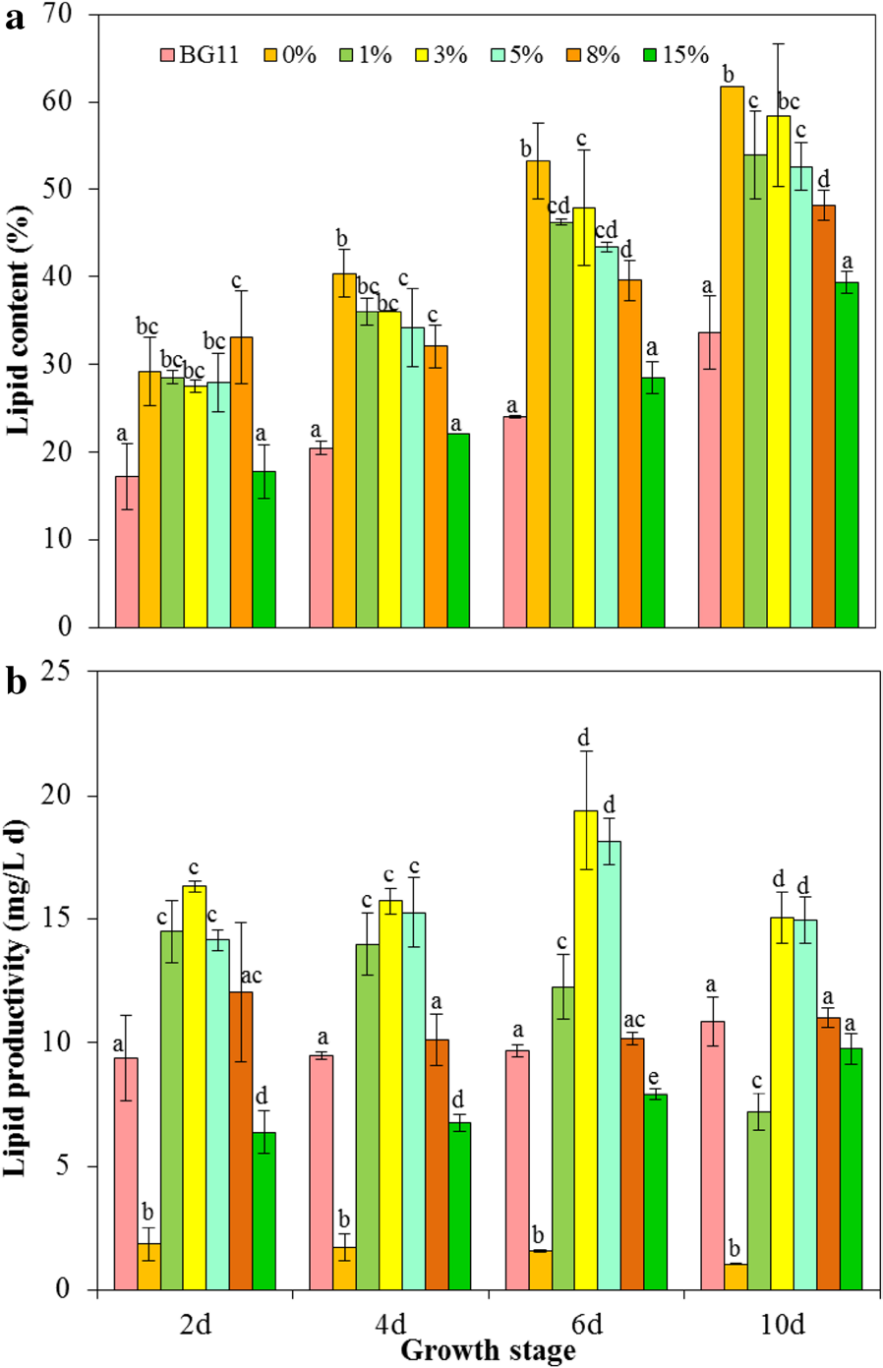
Lipid content (**a**) and lipid productivity (**b**) for the whole cultivation period of *Chlorella sorokiniana* SDEC-18 cells grown in BG11 and seawater supplemented with different volume percentages (0, 1, 3, 5, 8, and 15%) of anaerobically digested effluent from kitchen waste. *Data for the same growth stage followed by different letters are significantly different by Duncan’s test at *p* < 0.05

Figure 2a presents the oil gather quantitatively in the form of lipid content in algae cells under different ratio of seawater and ADE-KW and different growth stage. With rising proportions of seawater in the medium, the lipid contents of the algae were enhanced, which were all higher than that in BG11 medium. With increasing on growth time, algae also can accumulate more lipids. This was especially true in seawater, 1 and 3% ADE-KW, where the lipid content could reach about 60% at the 10th day, almost twice as high as that in the BG11 control. Algae with this value in lipid content (60%) outperformed many biodiesel production candidates, as Ajjawi et al. suggested [24]. Moreover, *C. sorokiniana* SDEC-18 got this lipid property in an easy way, just needing seawater and a little of wastewater (1–3%).

**Fig. 3:**
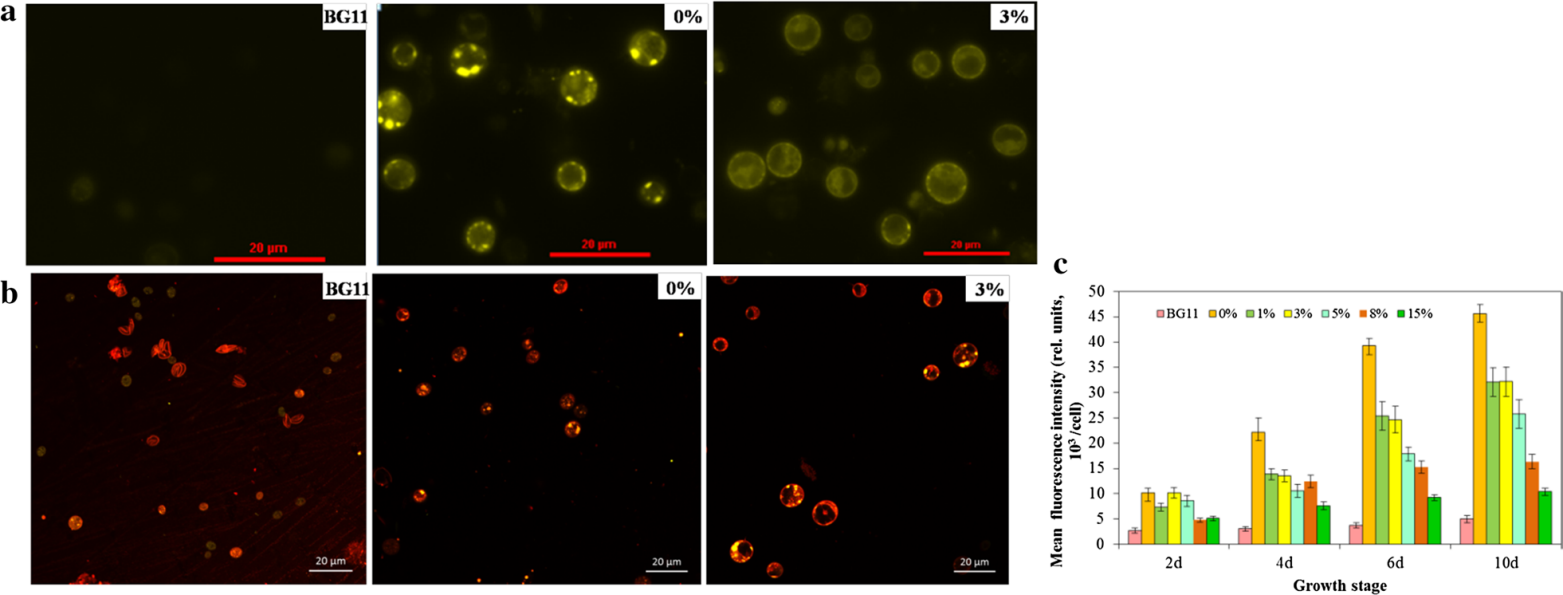
Images under **(a)** a fluorescence microscope and **(b)** a confocal microscope, and fluorescence intensity (**c**) for Nile Red-stained neutral lipid in *Chlorella sorokiniana* SDEC-18 cultivated in BG11 and seawater supplemented with different volume percentages (0 and 3%) of anaerobically digested effluent from kitchen waste. Shown in Fig. 3b are hydrocarbon oils stained using the neutral lipid-binding stain Nile Red (yellow) and chlorophyll autofluorescence (red). An individual cell was defined by chlorophyll autofluorescence from the chloroplast in each cell. Each image is an overlay of Nile Red signal, chlorophyll autofluorescence signal, and a bright-field image. Scale bar, 20 μm

**Fig. 4:**
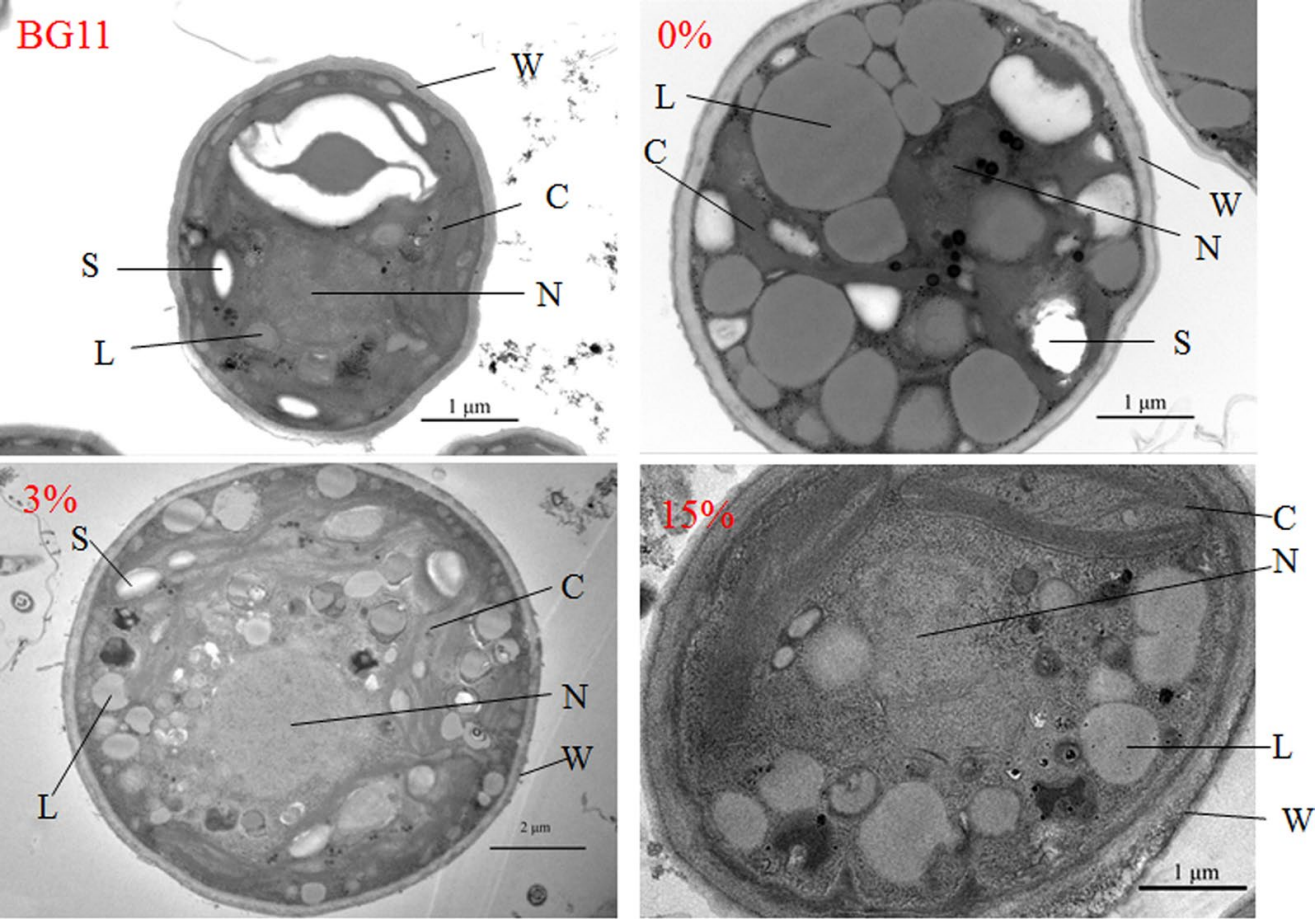
Cell ultrastructure of *Chlorella sorokiniana* SDEC-18 cells grown in BG11 and seawater supplemented with different volume percentages (0, 3, and 15%) of anaerobically digested effluent from kitchen waste. L, lipid droplets; S, starch granules; C, chloroplast; N, nucleolus; W, cell wall

The fluorescence intensity of Nile Red-stained neutral lipid visualized the lipid accumulation of the microalgae *C. sorokiniana* SDEC-18 under various ADE-KW loadings in seawater (Additional file 1: Figure S1). In the presence of DMSO, Nile Red dye penetrated into the cell and bounded itself to neutral lipids, thus making neutral lipids visualisable as yellow‒gold fluorescence under blue light excitation. Figure 3 qualitatively and quantitatively describes the lipid content of SDEC-18 grown in BG11, 0, and 3% ADE-KW which are representative, because BG11 and 0% ADE-KW were employed as control media, whereas 3 and 5% ADE-KW was excellent media with respect to growth rate. The fluorescence of SDEC-18 in 3% ADE-KW and 0% ADE-KW was obviously brighter than that in BG11 medium. The oil droplets in the ultrastructure of the cells shown in Fig. 4 convey the same message as the images produced by a confocal microscopy. For algae in 0% ADE-KW, big oil droplets dominated the cell structure, while the maximum and sum area of oil droplets in algal cell decreased with the ADE-KW concentration increasing.

High salinity from seawater must play an important role in triggering lipid accumulation in algal cells. Salt stress is an efficient method to enhance lipid yield of freshwater algae through shifting the carbon partitioning towards neutral lipids [10, 25, 26]. Nutrients starvation might also make some contributions.

Algae in seawater supplemented with 0–3% ADE-KW were also good at storing neutral lipid, reflected by fluorescence intensity in Fig. 3c. It is worth mentioning that the fluorescence intensity of SDEC-18 by the 10th day in 0% ADE-KW—namely 45.69 × 10^3^/cell—was 9 times higher than in BG11 medium, which also attained a statistically significant advantage over results in other groups (*p* < 0.01). Regarding the differences in intensity among all experimental groups, higher ADE-KW proportions led to lower fluorescence intensities. More nutrients were introduced into the algal cultivation system with higher ADE-KW proportions, which mitigated starvation stress, so it is the combination of high salinity and nutrients starvation enhanced algal lipid production to 60%.

Another interesting phenomenon is that there was very little neutral lipid increment in algal cells in BG11, while algae stored more and more neutral lipid with on-going cultivation time under the stress caused by seawater and the ADE-KW wastewater. Moreover, algae in all media containing seawater started to synthesize neutral lipid quickly during their fast growth phase, around the 6th day. This did not occur in BG11.

#### Relationship between algae growth and lipid accumulation

As Williams and Laurens reported, an inverse relationship between lipid increase and biomass loss is common [27]. Compared to algae cultured in BG11, the relationships between biomass loss and lipid increase for algae living in media prepared with seawater are separated into three categories: (1) the increase in lipid content exceeded the decrease in growth, resulting in higher lipid content and productivity, as in 3 and 5% ADE-KW; (2) the decrease in growth exceeded the increase in lipid synthesis, resulting in lower lipid productivity, as with 0 and 1% ADE-KW; and (3) the increase in lipid content was balanced by the decrease in growth, resulting in practically stable lipid productivity in 8 and 15% ADE-KW. In this study, the adverse growth conditions mainly caused biomass loss, including nutrient deficiency and salinity, which are common for stimulation of lipid accumulation in algae.

Figure S2 (in Additional file 1) contains data for growth rate derived from biomass concentration and the corresponding lipid content, and an analysis of the relationship between them. The data are scattered; however, an inverse relationship between growth rate and lipid content is present (*R*^2^ = 0.49) (Additional file 1: Figure S2a).

#### Lipid productivity

For microalgae-based biofuel production that concerns biomass and lipid, lipid productivity is a great parameter to evaluate the potential of algae for biodiesel production. It is defined as the product of lipid content (mg/L) and growth rate (day^−1^) to give a lipid-normalized production rate. Our results with ADE-KW in seawater suggested that lipid productivity follows a quadratic relationship with growth rate (*R*^2^ = 0.46), manifesting an intermediate maximum. The red spot in Figure S2b (in Additional file 1) is the maximum lipid production rate (14.96 mg/L  day) calculated from the first derivative of the quadratic equation, corresponding to a growth rate of 0.41/day. This datum is close to the points denoting 3 and 5% media on the 4th day, which indicated a lipid accumulation at a high speed.

Rather than harvesting algae as usual in their stationary phase, for lipid production, it is more appropriate, economical, and even efficient to collect algae grown in 3 or 5% ADE-KW during the exponential phase, due to the lipid productivities of 19 and 18 mg/L  day observed on the 6th day (Fig. 2b). The productivities are about 1.8 times as high as the highest 11 mg/L  day attained in BG11 medium on the 10th day (*p* < 0.05). Our findings indicated that seawater supplemented with ADE-KW was an ideal medium for *C. sorokiniana* SDEC-18 to produce biodiesel economically and efficiently.

#### Fatty acid profile

The alterations to algal lipids caused by seawater and ADE-KW include not just lipid content, but also FA profiles. Table S2 (in Additional file 1) presents the fatty acid (FA) profiles of *C. sorokiniana* SDEC-18 in BG11 and in media prepared with seawater and ADE-KW. The fatty acids in SDEC-18 cells included saturated fatty acids (SFA: palmitic C16:0), monounsaturated fatty acid (MUFA: palmitoleic C16:1, oleic C18:1), and polyunsaturated fatty acid (PUFA: hexadecadienoic C16:2, linoleic C18:2, arachidonic, C20:4, docosatetraenoioc C22:4).

Except the 8% ADE-KW medium, algae in other media containing seawater exhibit lower SFA proportion than that in BG11. However, environmental stress usually induced a cascade of reactions, leading to the formation of acetyl-CoA, which would induce more synthesis of saturated fatty acids. This unconformity might result from further desaturation and elongation reactions of these saturated fatty acids, which also contributed to the higher apparent MUFA levels of algae in seawater than in BG11. The MUFA proportion increased from 34 to 51% with increase in seawater volume from 90 to 100% (0% ADE-KW to 10% ADE-KW).

About PUFA, it decreased in all media with seawater compared with that in BG11. Lower PUFA content can result from nitrogen and phosphorus limitation, as the theory of Cheng et al. [28], and can also be obtained by salt stress, because microalgae need to reduce membrane fluidity and permeability to achieve a rigid cell and an osmotic balance to cope with high salinity [25, 26]. Van Eerden et al. [29] reported that the fluidity of the membrane is attributed to the presence of polyunsaturated lipid tails, so compared to BG11, lowering PUFA in other media is a positive reaction to facilitate survival of algae in seawater and ADE-KW. That also demonstrated that the alga SDEC-18 has a powerful ability to adapt to harsh environments and could be used as feedstock for biofuel with low investment.

### Settling efficiency

Another bottleneck for commercialization of microalgae-based industrial processes is high-cost harvesting. There were much research which has been conducted into different methods for cutting down the energy input in bulk harvesting process [30]. Fortunately, some algae species are known to flocculate spontaneously without any chemical addition [31]. This spontaneous flocculation process, termed as autoflocculation, is a promising economical and eco-friendly harvesting method for microalgae. As a result, the aggregate can be easily harvested by sedimentation.

**Fig. 5:**
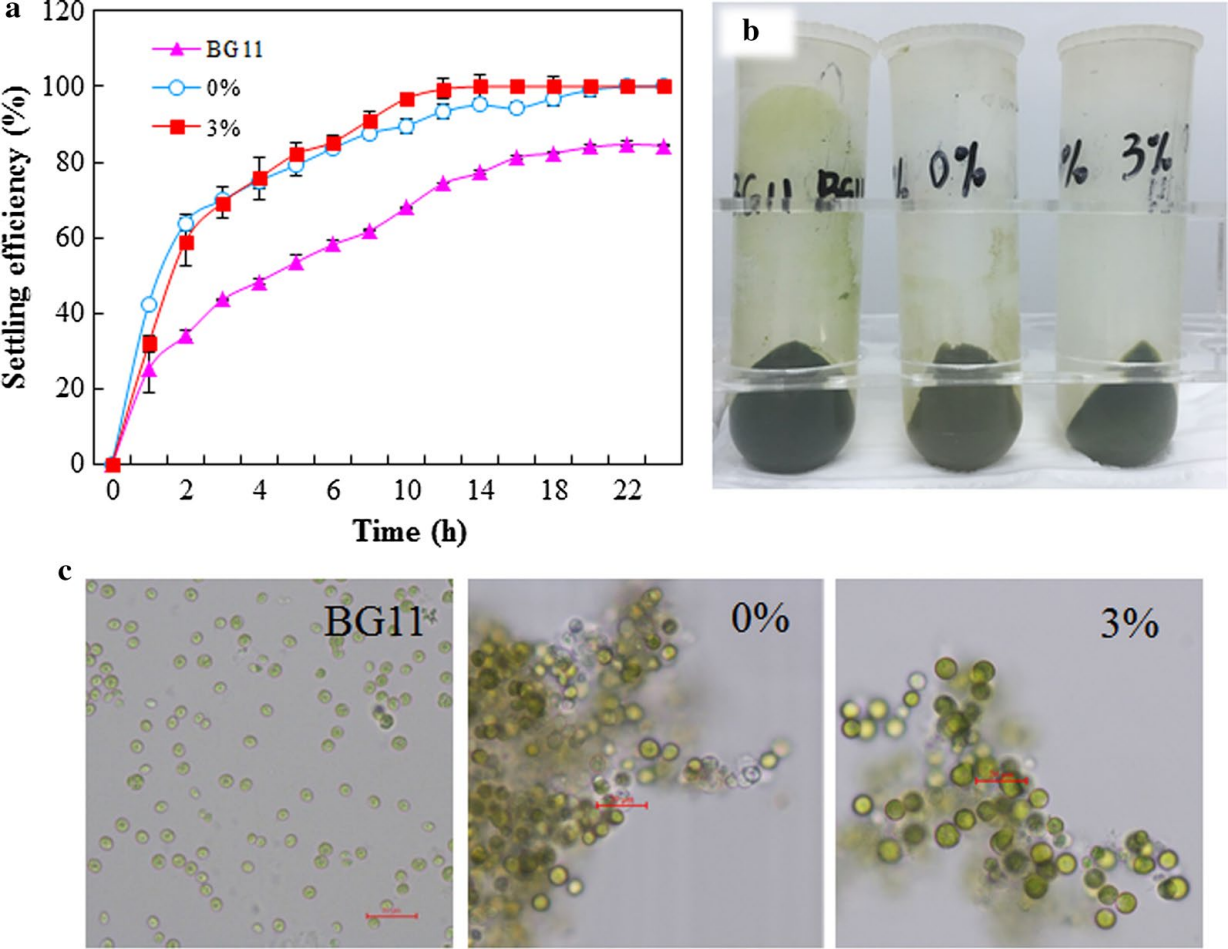
Settling efficiency of *Chlorella sorokiniana* SDEC-18 in BG11, and in seawater with 0 and 3% anaerobically digested effluent from kitchen waste after 10-day cultivation. **a** Settling efficiency. **b** Smearing phenotype in centrifuge bottle. **c** Appearance of flocs in algal cultures. Scale bar, 20 μm

*Chlorella sorokiniana* SDEC-18 exhibited a good ability to sediment, as shown in Fig. 5a. It needs just 5 h to get a harvesting efficiency of approximately 80% in media prepared with seawater, and after about 8 h, more than 90% of the biomass settled at the bottom. However, there was no apparent positive harvesting effect in culture from BG11 medium, *which only attained* 50% settled at the 5th hour and 60% at the 8th hour. Moreover, smearing, which lowers the harvest efficiency, did not occur in the centrifuge bottles for harvest of algae in 0 and 3% ADE-KW, but did appear for BG11 medium (Fig. 5b).

Regarding the higher settling efficiency in media containing seawater, charge neutralization is a key mechanism of aggregation, i.e., the attraction between the negatively charged algal cell surface and the positively charged matter [27]. If an excess of calcium ions is present, the calcium phosphate precipitate will be positively charged and algal cells serve as a solid support for the precipitate, and then, charge neutralization is accomplished [31]. The kinds and concentrations of metal elements in seawater (such as calcium, aluminum, and magnesium) are more numerous and higher than those in BG-11 medium (Additional file 1: Table S1). Moreover, the pH values in the three media exceed 9, due to photosynthetic CO_2_ depletion (data not shown), which can cause supersaturation of ions, such as calcium, magnesium, and phosphate. Then, these calcium or magnesium precipitates carrying positive surface charges induced flocculation through charge neutralization and/or sweep coagulation.

Microalgae in media other than BG11 flocculated together to give rise to flocs large enough to be seen by the naked eye, and were surrounded by transparent colloidal material (Fig. 5c). Moderate cell aggregation and the appearance of cell clusters also occurred in *Nannochloropsis* sp. cultures that encountered a stress of high NaCl concentration and illumination [32]. In our study, stressful conditions caused by high salinity and nutrient deprivation from seawater can assist in forming these colonies in clusters. Thus, cultivation of algae with seawater could easily achieve good settling capability for algae without complicated and expensive treatment.

### Response of algae to seawater combined with ADE-KW

Confronting seawater and seawater modified by ADE-KW in five concentrations (1, 3, 5, 8, and 15%), the responses of *C. sorokiniana* SDEC-18 in morphology, membrane permeability, and photosynthetic system were detected.

#### Cell morphology

**Fig. 6:**
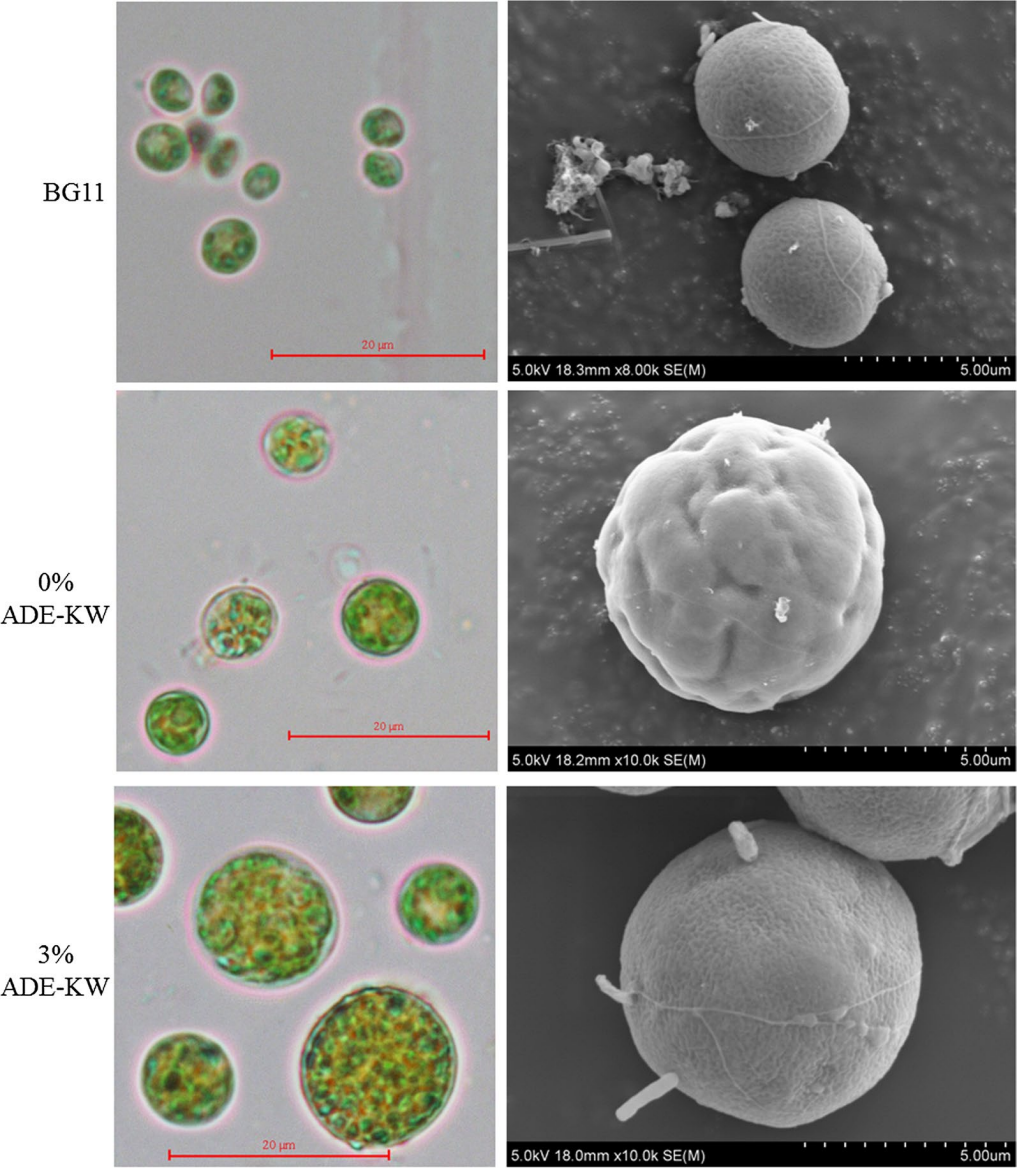
Morphological observation of *Chlorella sorokiniana* SDEC-18 under an optical microscope (left column) and a scanning electron microscope (right column) for the tested algae cultivated in BG11 and in seawater supplemented with 0 and 3% by volume percentages of anaerobically digested effluent from kitchen waste (ADE-KW)

**Fig. 7:**
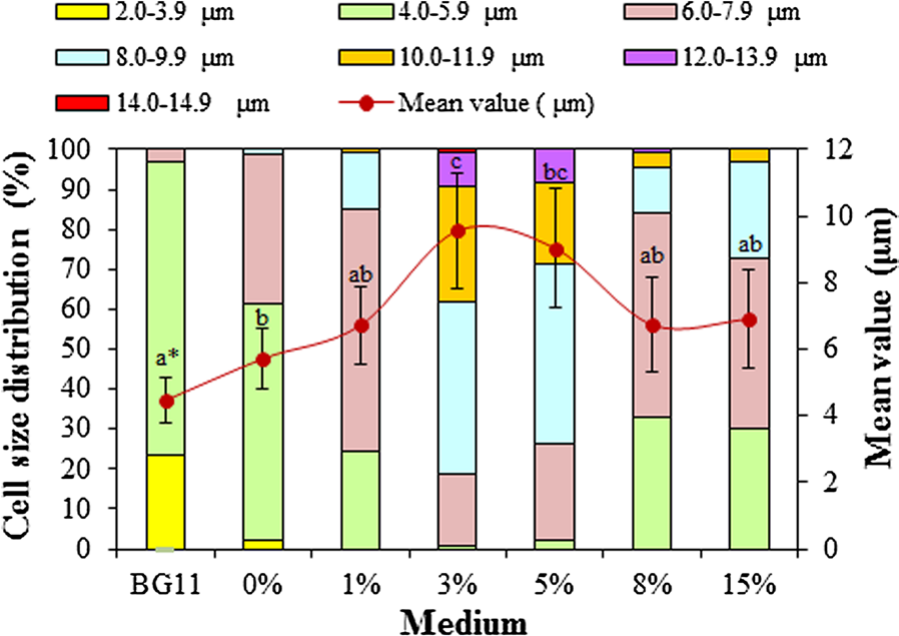
Cell diameters of *Chlorella sorokiniana* SDEC-18 cells grown in BG11 and seawater supplemented with different volume percentages (0, 1, 3, 5, 8, and 15%) of anaerobically digested effluent from kitchen waste. *Data followed by different letters are significantly different by Duncan’s test at *p* < 0.05

Aiming at cell morphology change under seawater and ADE-KW, we observed the algal surface (Fig. 6) and calculated the cell size (Fig. 7). Under a microscope, SDEC-18, in standard freshwater medium-BG11, is spherical with a diameter of 2–6 µm, without flagellum, and has a characteristic emerald-green color. However, the cell wall of algae in pure seawater became obviously wrinkled into irregular folds (Fig. 6, 0% ADE-KW). Savchenko et al. [33] also reported a similar physiological change of microalgae cell under shear stress from ultrasound. This change increased cell-specific surface area for nutrient uptake and may be considered as a protect reaction under stress including salinity, nutrient limitation, and mechanical force. However, the surface of algae cell in 3% ADE-KW became less wrinkled and looked more similar with that in BG11, compared with cell in 0% ADE-KW (Fig. 6). The phenomenon suggested a mitigation by ADE-KW to stresses imposed on algae by seawater; indeed, the specific mechanism needed a further research.

Figure 7 shows the cell size distribution of SDEC-18 and demonstrates the significantly lengthened cells that exist in seawater and/or ADE compared with those in BG11. *Chlorella sorokiniana* SDEC-18 has a mean diameter of 5.72 μm in seawater, while in 3% ADE-KW, an amazing datum was observed with 9.56 μm in mean diameter, which is 1.7 times larger than cells in BG11 (mean diameter of 4.47 μm). In BG11 and seawater, the range of algal cell diameters of 4.0‒5.9 μm accounted for the most cells present, but in seawater supplemented with ADE-KW, 6.0‒9.9 μm cells dominated the population. Especially in the 3% ADE-KW, the maximum diameter of the cells observed was 14.56 μm, which is possibly a stress response.

Ultrastructural images in Fig. 4 and electron microscopy photos in Fig. 6 also revealed the enlarged cells of SDEC-18 in cultures with seawater. With respect to microalgal cell density, the cell numbers in media with seawater were much lower than in BG11 (Table 1), which indicates a reduction in cell division. Arrest in cell division, perhaps, gave more time to cell enlargement for the algae and caused an increase in cell size, resulting in increased mass per cell (Table 1). Under seawater salinity governed by NaCl, Kim et al. [10] observed a decreased cell count and an increased cell size of *C. sorokiniana* HS1, with subsequently increased dry cell mass. Jiang and Chen also studied the effects of salinity on microalgae cell growth and observed inhibited growth and elongated cells at extremely high NaCl concentrations [34].

#### Membrane permeability

The cytoplasm of microalgal cells is surrounded by a layer of cell membrane that possesses unique features of selective permeability. Material, energy, and information transfer between the cells and the environment must be carried out through the cell membrane. However, too high, an osmotic pressure in the external environment is bound to affect the integrity of cell membranes and normal physiological function. In this study, relative electrical conductivity was calculated to represent the membrane permeability.

**Fig. 8:**
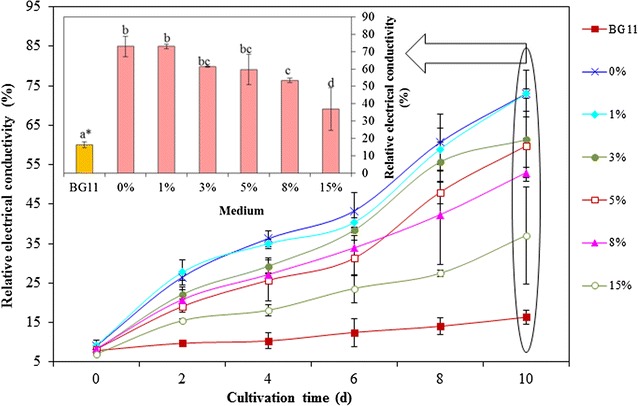
Relative electrical conductivity of *Chlorella sorokiniana* SDEC-18 grown in BG11 and seawater supplemented with different volume percentages (0, 1, 3, 5, 8, and 15%) of anaerobically digested effluent from kitchen waste. The final relative electrical conductivity is summarized in the inset. *Data followed by different letters are significantly different by Duncan’s test at *p* < 0.05

With variation of the ADE-KW addition ratio from 15 to 0%, salinity in the medium increased. Figure 8 indicates that microalgae cell membrane permeability increased with the increase of salinity. The continuous leakage with cultivation period (Additional file 1: Figure S2b) demonstrated that the membrane damage caused by high osmotic pressure was irreversible. Since mild or strictly localized degrees of cell membrane damage might allow fluxes of ions such as Ca^2+^ and Mg^2+^ sufficient to trigger the mechanism leading to apoptosis [35], membrane permeability increases caused by high salinity may be responsible for the death of microalgae. Our results suggested that when *C. sorokiniana* SDEC-18 was cultivated in pure seawater, the membrane was damaged severely, as depicted in ultrastructural images (Fig. 4) and relative electrical conductivity measurements.

The relative electrical conductivity in the microalgal suspension increased under seawater cultivation, which indicated that electrolytes leaked from the microalgal cells. That is to say, the microalgal cell membranes were damaged when they were exposed to seawater for several days. Almost no growth was observed for SDEC-18 at the extremely high NaCl concentration of pure seawater: growth was inhibited, and the cells tended to undergo apoptosis. At other ratios of ADE, the microalgae also did not grow as ideally as in BG11 medium.

#### Photosynthetic system

High salinity leads to multiple biological and biophysical changes of algae that can cause cell death, due specifically to osmotic stress and damage to photosystem II (PS II) in photosynthetic freshwater microorganisms [36, 37]. Inhibited photosynthesis and cell division also occurred to the freshwater microalga *Botryococcus braunii* under high-level NaCl addition [38]. The salt-induced cellular change is an enhanced accumulation of reactive oxygen species (ROS), ultimately imposing a secondary oxidative stress [39, 40]. In photosynthetic organisms, ROS can disturb normal metabolism and decrease photosynthetic efficiency through the peroxidation of lipids in the thylakoids and the damage to the PS II complex by destroying the electron transport chain [41, 42]. Trace amounts of nitrogen and phosphorus in seawater cannot satisfy algal photosynthesis, respiration, and synthesis of nitrogenous compounds like protein, chlorophyll, and DNA. Degradation or reduction in photosynthetic pigments like Chl a is a common stress response in plants and microalgae [43]. The above-mentioned factors explain the far lower concentration of Chl a in seawater than in BG11, and the increased concentration promoted by nutrients in the ADE-KW added to seawater (*p* < 0.05, Additional file 1: Table S3).

All the pigments other than Chl a (mainly carotenoids) protect photosynthetic assemblies from photosensitization processes, and ratios of Chl a/Chl b and carotenoids/(Chl a + Chl b) reflect the PS II activity [44, 45]. Compared with BG11 medium, decrease in the Chl a/Chl b ratio indicates a damaged photosynthetic system, which is also suggested by the increment in the carotenoids/(Chl a + Chl b) ratio, and this indirectly includes PS II depression and oxidative stress. Pancha et al. [46] also observed decrease in Chl a and increment in carotenoids/Chl a + Chl b under nitrogen starvation for diminishing light-harvesting complex and PS II activity. Salinity brought high ratios in carotenoids/(Chl a + Chl b) to reduce the antenna size, which is a protective function against photo-oxidative damage [25].

Compared to algae cultured in BG11, higher values of Chl a/Chl b and lower values of carotenoids/Chl a + Chl b in media consisting of seawater and ADE-KW indicate that *C. sorokiniana* SDEC-18 can trigger a self-protective system and adapt to environmental stress. The alga SDEC-18 is robust for culture variation, which is a characteristic of the ideal microalgae described by Wijffels and Barbosa [3]. Moreover, addition of ADE-KW is an efficient way to facilitate algal viability when seawater is used as the culture medium.

## Conclusion

The stresses from high salinity and scarce essential nutrients in seawater benefited lipid accumulation in algal cells, but with low lipid productivity (< 2 mg/L  day), as the membrane permeability and photosynthetic system were damaged. Fortunately, ADE-KW mitigated inhibition from stress and the highest lipid productivity (19 mg/L  day) was obtained when 3 and 5% ADE-KW was added to seawater, almost double of 10.55 mg/L  day in BG11 medium.

Besides increased lipid production efficiency, seawater with ADE-KW also guaranteed the alga SDEC-18 two other desirable merits for microalgae producing biofuel, enlarged cells, and increased settling efficiency. In a word, seawater added a glug of ADE-KW can be a reliable and promising method to cultivate limnetic microalgae for fast lipid production in a cost-effective way.

## Additional file

**Additional file 1: Table S1.** Compositions of BG11, seawater, and anaerobically digested effluent from kitchen waste (ADE-KW). **Table S2.** Fatty acid profiles obtained from *Chlorella sorokiniana* SDEC-18 (as percentage of total fatty acid methyl esters (FAME)). **Table S3.** The final concentration of Chl a, ratio of Chl a/Chl b, and Carotenoids/(Chl a + Chl b) for *Chlorella sorokiniana* SDEC-18 grown in BG11 and in seawater supplemented with different volume percentages (0, 1, 3, 5, 8 and 15%) of anaerobically digested effluent from kitchen waste. **Figure S1.** Neutral lipid accumulation in *Chlorella sorokiniana* SDEC-18 cultivated in BG11 and seawater supplemented with different volume percentages (0, 1, 3, 5, 8 and 15%) of anaerobically digested effluent from kitchen waste. Shown are hydrocarbon oils stained using the neutral lipid-binding stain Nile Red (yellow) under a fluorescence microscope. Scale bar, 20 μm. **Figure S2.** The relationships of growth rate with lipid content (a), and growth rate with lipid productivity (b). The red square in graph b stands for the maximum lipid production rate calculated from the first derivative of the quadratic equation.
