# Supplementary material for: Lipid productivity in limnetic Chlorella is doubled by seawater added with anaerobically digested effluent from kitchen waste
Source: Biotechnol Biofuels. 2018 Mar 14;11:68. doi: 10.1186/s13068-018-1064-5 (PMC5851330; doi:10.1186/s13068-018-1064-5)
Supplement: Supplementary file 1 — Additional file 1: Table S1. Compositions of BG11, seawater, and anaerobically digested effluent from kitchen waste (ADE-KW). Table S2. Fatty acid profiles obtained from Chlorella sorokiniana SDEC-18 (as percentage of total fatty acid methyl esters (FAME)). Table S3. The final concentration of Chl a, ratio of Chl a/Chl b, and Carotenoids/(Chl a + Chl b) for Chlorella sorokiniana SDEC-18 grown in BG11 and in seawater supplemented with different volume percentages (0, 1, 3, 5, 8 and 15%) of anaerobically digested effluent from kitchen waste. Figure S1. Neutral lipid accumulation in Chlorella sorokiniana SDEC-18 cultivated in BG11 and seawater supplemented with different volume percentages (0, 1, 3, 5, 8 and 15%) of anaerobically digested effluent from kitchen waste. Shown are hydrocarbon oils stained using the neutral lipid-binding stain Nile Red (yellow) under a fluorescence microscope. Scale bar, 20 μm. Figure S2. The relationships of growth rate with lipid content (a), and growth rate with lipid productivity (b). The red square in graph b stands for the maximum lipid production rate calculated from the first derivative of the quadratic equation. [file 13068_2018_1064_MOESM1_ESM.docx]

**Supplementary information**

**Lists:**

**Table S1** Compositions of BG11, seawater, and anaerobically digested effluent from kitchen waste (ADE-KW).

**Table S2** Fatty acid profiles obtained from *Chlorella* *sorokiniana* SDEC-18 (as percentage of total fatty acid methyl esters (FAME)).

**Table S3** The final concentration of Chl a, ratio of Chl a / Chl b, and Carotenoids / (Chl a + Chl b) for *Chlorella* *sorokiniana* SDEC-18 grown in BG11 and in seawater supplemented with different volume percentages (0, 1, 3, 5, 8 and 15%) of anaerobically digested effluent from kitchen waste.

**Fig. S1** Neutral lipid accumulation in *Chlorella* *sorokiniana* SDEC-18 cultivated in BG11 and seawater supplemented with different volume percentages (0, 1, 3, 5, 8 and 15%) of anaerobically digested effluent from kitchen waste. Shown are hydrocarbon oils stained using the neutral lipid-binding stain Nile Red (yellow) under a fluorescence microscope. Scale bar, 20 μm.

**Fig. S2** The relationships of growth rate with lipid content (a), and growth rate with lipid productivity (b). The red square in graph b stands for the maximum lipid production rate calculated from the first derivative of the quadratic equation.

**Table S1** Compositions of BG11, seawater, and anaerobically digested effluent from kitchen waste (ADE-KW).

| Composition | BG11^*^ | Seawater | ADE-KW |
| --- | --- | --- | --- |
| TN (mg/L) | 247.06 | 3.67 ± 0.14 | 2158.62 ± 53.67 |
| NO_3_-N (mg/L) | 247.06 | _ | 33.44 ± 1.20 |
| NH_3_-N (mg/L) | 0.517 | 0.601 ± 0.002 | 2003.83 ± 47.77 |
| TP (mg/L) | 7.12 | 0.0027 ± 0.00 | 20.24 ± 0.11 |
| PO_3_-P (mg/L) | 7.12 | _ | 11.43 ± 0.71 |
| Na (mg/L) | 414.76 | 13920 ± 142 | 4478 ± 21 |
| K (mg/L) | 17.93 | 346.6 ± 7.2 | 1478.4 ± 31.6 |
| Ca (mg/L) | 9.80 | 988.8 ± 13.6 | 135.08 ± 0.28 |
| Mg (mg/L) | 7.317 | 1295 ± 3 | 86.94 ± 0.44 |
| Al (mg/L) | _ | 24.1 ± 3.58 | 1.876 ± 0.01 |
| Fe (mg/L) | 0.689 | 5.6 ± 0.08 | 1.486 ± 0.04 |
| Cu (mg/L) | 0.02 | 5.2 ± 0.06 | 1.212 ± 0.085 |
| Zn (mg/L) | 0.05 | 2.61 ± 0.08 | 1.146 ± 0.004 |
| Mn (mg/L) | 0.517 | _ | _ |
| Si (mg/L) | _ | 78.65 ± 0.34 | _ |
| Sr (mg/L) | _ | 9.92 ± 0.03 | _ |
| Mo (mg/L) | 0.155 | _ | 0.308 ± 0.038 |
| Co (mg/L) | 0.01 | _ | _ |
| TOC (mg/L) | _ | _ | 3761.55 ± 15.86 |

^*^ The data in this column are calculated based on the components of BG11. _ Not detected.

**Table S2** Fatty acid profiles obtained from *Chlorella* *sorokiniana* SDEC-18 (as percentage of total fatty acid methyl esters (FAME)).

| FAME composition |  | BG11 | 0% | 1% | 3% | 5% | 8% | 15% |
| --- | --- | --- | --- | --- | --- | --- | --- | --- |
| SFA | C16:0 | 22.95 | 18.99 | 18.27 | 17.75 | 20.65 | 27.53 | 19.36 |
|  | Subtotal | 22.95 | 18.99 | 18.27 | 17.75 | 20.65 | 27.53 | 19.36 |
| MUFA | C16:1 | - | 3.00 | 2.90 | 3.55 | 2.63 | 3.47 |  |
|  | C18:1 | 21.61 | 47.25 | 48.24 | 42.42 | 33.88 | 28.52 | 21.13 |
|  | Subtotal | 21.61 | 50.25 | 51.14 | 45.97 | 36.51 | 31.99 | 21.13 |
| PUFA | C16:2 | - | - | - | - | - | 6.32 | - |
|  | C18:2 | 19.67 | 12.66 | 13.45 | 11.81 | 12.86 | 14.08 | 14.14 |
|  | C20:4 | - | - | 2.36 | - | - | 4.06 | - |
|  | C22:4 | 17.37 | 10.35 | - | 14.01 | 18.74 | - | 12.82 |
|  | Subtotal | 37.04 | 23.01 | 15.81 | 25.82 | 31.60 | 24.46 | 26.96 |

SFA = saturated fatty acids.

MUFA = monounsaturated fatty acids.

PUFA = polyunsaturated fatty acids.

**Table S3** The final concentration of Chl a, ratio of Chl a / Chl b, and Carotenoids / (Chl a + Chl b) for *Chlorella* *sorokiniana* SDEC-18 grown in BG11 and in seawater supplemented with different volume percentages of anaerobically digested effluent from kitchen waste.

| Medium | Chl a (mg/L) | Chl a / Chl b | Carotenoids / (Chl a + Chl b) |
| --- | --- | --- | --- |
| BG11 | 1.500 ± 0.213^a^* | 3.418 ± 0.488 | 0.192 ± 0.101 |
| 0% | 0.372 ± 0.076^b^ | 0.841 ± 0.047 | 1.139 ± 0.123 |
| 1% | 0.500 ± 0.009^bc^ | 1.056 ± 0.122 | 0.974 ± 0.092 |
| 3% | 0.581 ± 0.153^bc^ | 1.173 ± 0.028 | 0.878 ± 0.186 |
| 5% | 0.705 ± 0.117^bc^ | 1.672 ± 0.063 | 1.341 ± 0.282 |
| 8% | 1.058 ± 0.046^d^ | 2.410 ± 0.248 | 0.736 ± 0.035 |
| 15% | 1.169 ± 0.023^ad^ | 1.657 ± 0.137 | 0.715 ± 0.061 |

* Data in the same column followed by different letters are significantly different by Duncan’s test at *p* < 0.05.

**Lipid accumulation change**

The concentrations of some elements (including Na^+^, Ca^2+^, Mg^2+^ in Table A1) were much higher than the salt content in cells, thereby leading to a lower water potential in the medium than that in cell, and the loss of water. That contributed to the loss of water by penetration from the cells and finally decreased algal viability and growth rate [1]. The phenomenon might be defined as salt stress or osmotic stress, which is the adverse effect of excess soluble minerals [2]. The presence of excessive soluble salts in a medium interferes with the uptake and metabolism of essential mineral nutrients [3]. The double ratio of Na^+^/K^+^ in seawater compared to BG11 would lead to an increased Na uptake and then deficiency in potassium, nitrogen and calcium.

The other stress for triggering lipid accumulation might be rooted in the elements in seawater, which were all at a deficient condition compared to BG11 and might impact lipid accumulation as in starvation (such as nitrogen and phosphorus in Table A1). The nitrogen source in seawater was only equivalent to 1.48% of that in BG11. Although available nitrogen for algal growth increased as the volume of added wastewater was increased, still only about 100 mg/L nitrogen was present in 5% ADE-KW, which is half that of BG11. The deficiency of phosphorus was more apparent than nitrogen, with almost no P in seawater and limited P in ADE-KW. Those environmental stresses stimulated lipid synthesis mainly through disturbing the citric acid cycle, leading to citrate accumulation and subsequently to its excretion in the cytosol, as a precursor of acetyl-CoA, and then neutral lipid [4].

Fig. A2 depicts the increased lipid content and fluorescence intensity with algal growth under abovementioned factors.

The lipid content corroborated with Nile Red staining, suggested that this significant increase in lipid accumulation in media prepared with seawater might be rooted in the combination of salinity stress from nutritive salts (Na^+^, Ca^2+^, Mg^2+^, Al^3+^, and so on) and nutrient deficiency (TN, TP, NH_3_-N, and so on). So seawater and wastewater applied in algal cultivation could trigger *de novo* biosynthesis of lipid in an easy and economical way.


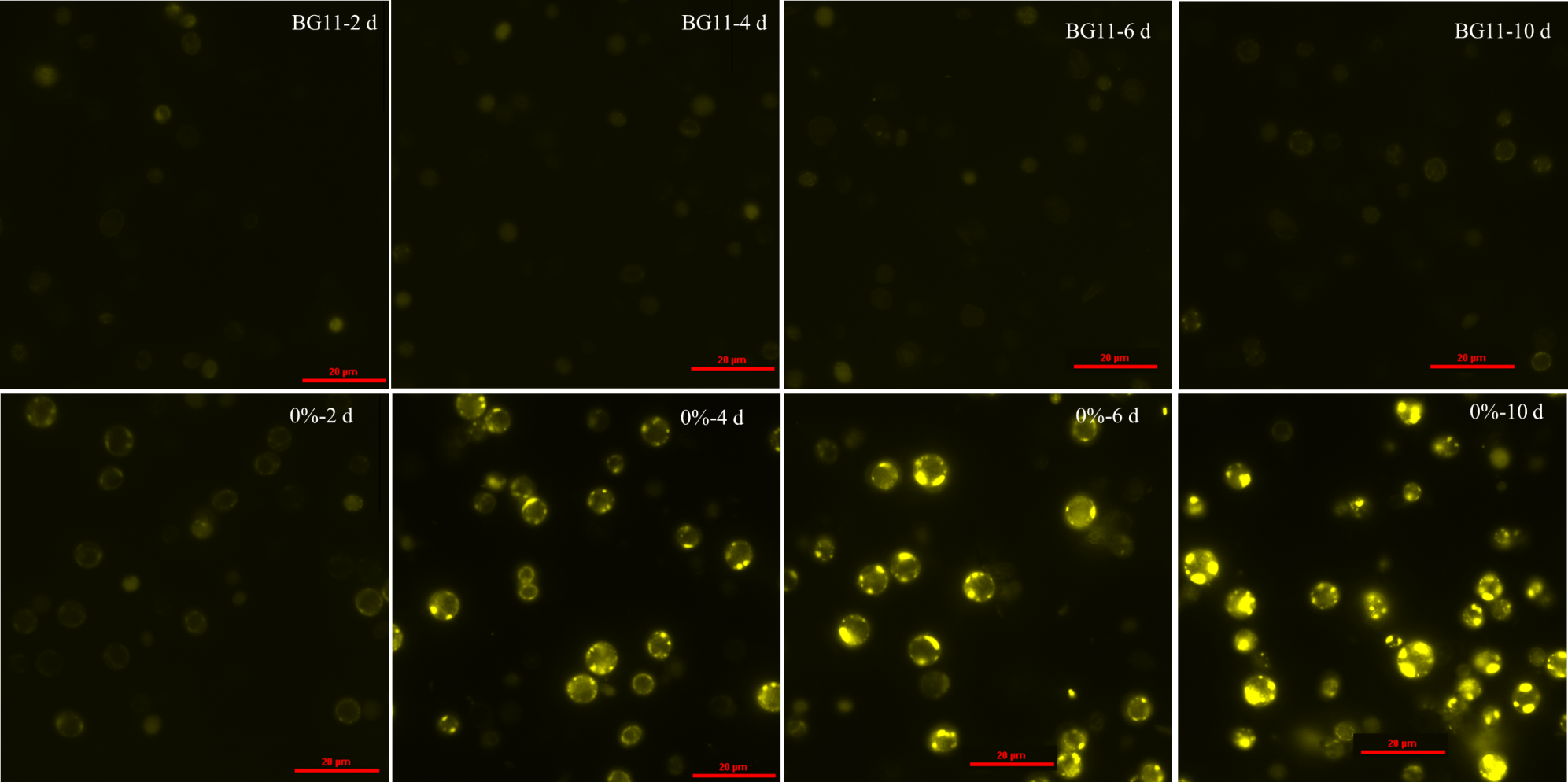


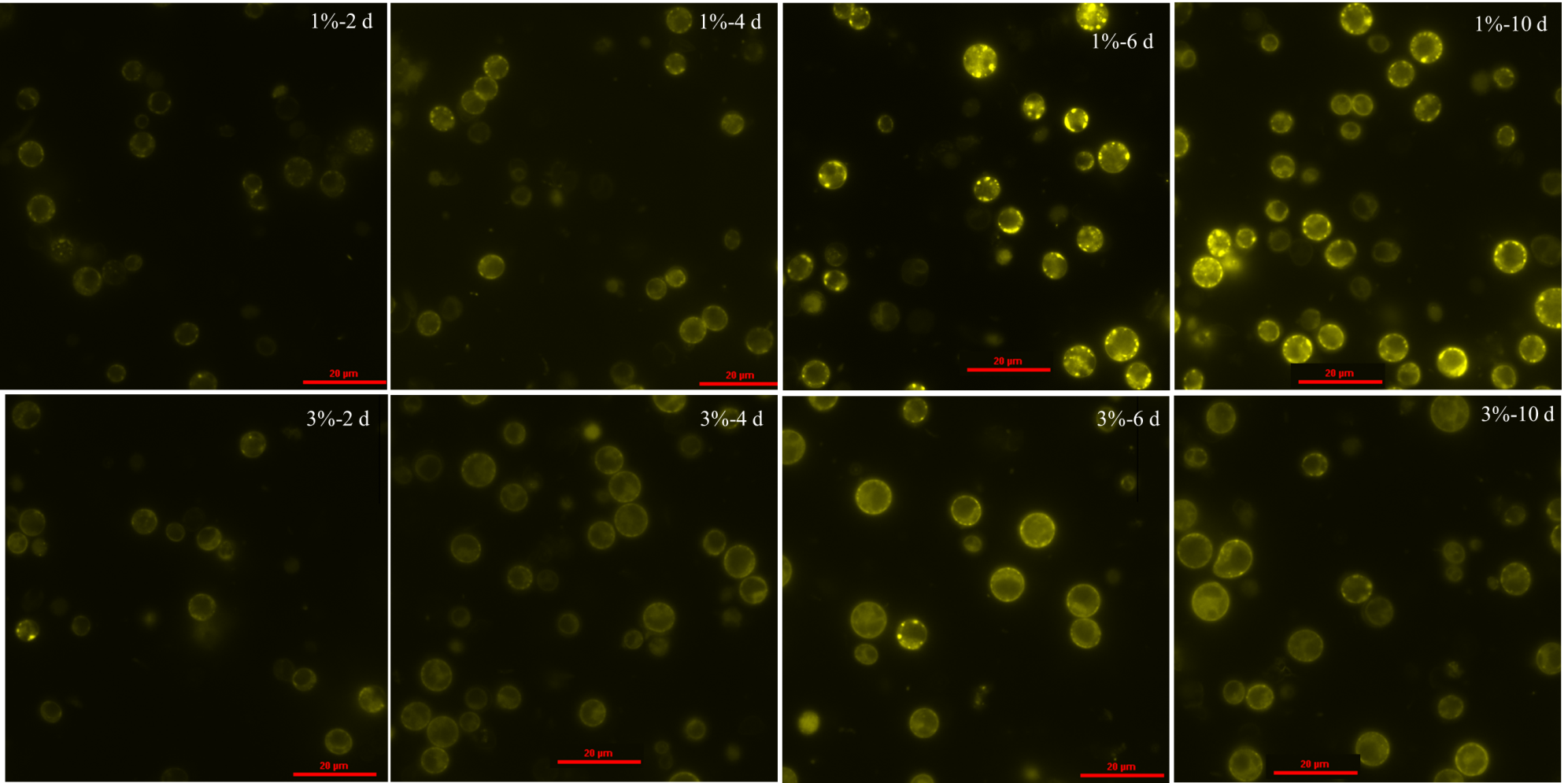

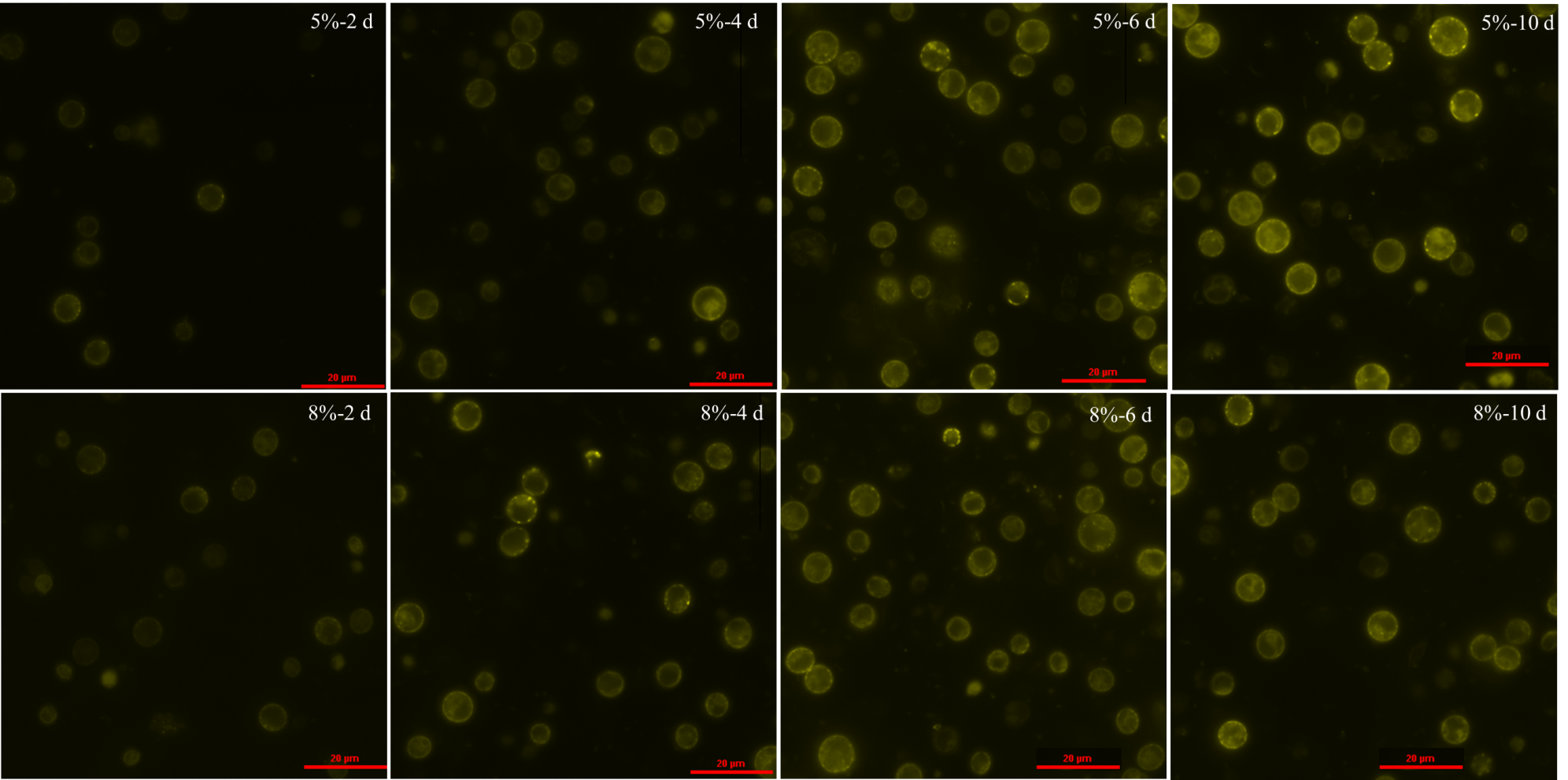

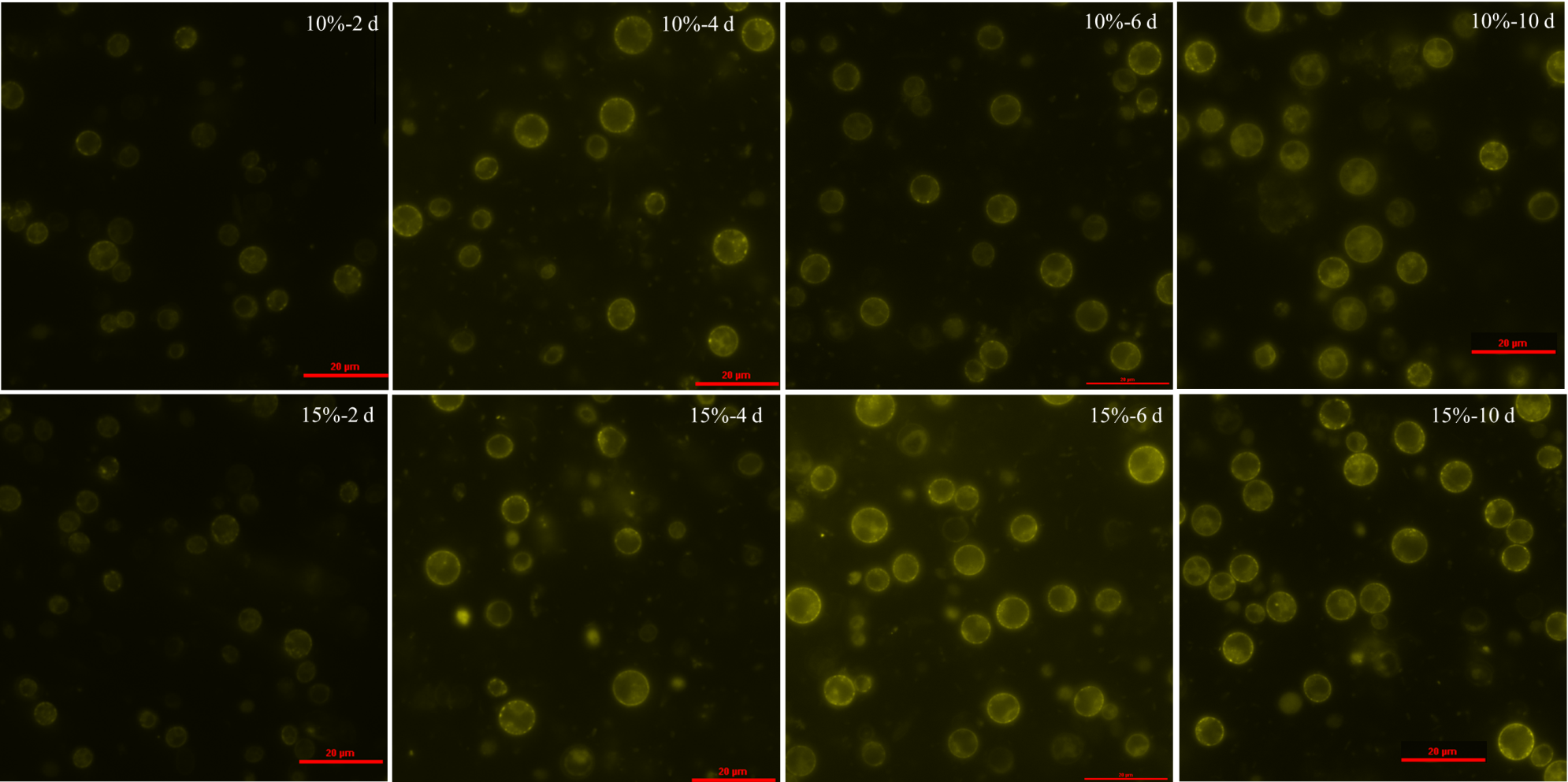


**Fig. S1** Neutral lipid accumulation in *Chlorella* *sorokiniana* SDEC-18 cultivated in BG11 and seawater supplemented with different volume percentages (0, 1, 3, 5, 8 and 15%) anaerobically digested effluent from kitchen waste. Shown are hydrocarbon oils stained using the neutral lipid-binding stain Nile Red (yellow) under a fluorescence microscope. Scale bar, 20 μm.

The caption on the figure presents the medium and sample point of cells, *e.g.* ‘BG11-2 d’ suggests the cells cultivated in BG11 and sampled at the 2^nd^ day.

**
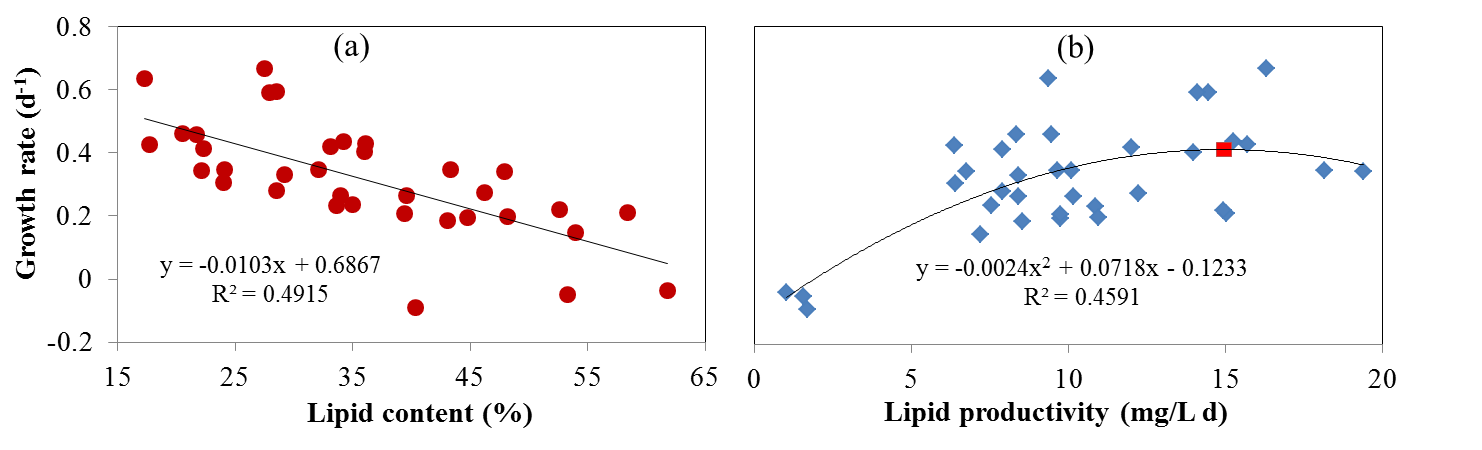
**

**Fig. S2** The relationships of growth rate with lipid content (a), and growth rate with lipid productivity (b). The red square in graph b stands for the maximum lipid production rate calculated from the first derivative of the quadratic equation.

**References**

1. Buchanan BB, Gruissem W, Jones RL. *Biochemistry & Molecular Biology of Plants*, American Society of Plant Physiologists, 2000, p.21–23, 369–376.
2. Munns R. Genes and salt tolerance: bringing them together. New Phytol. 2005;167:645–663.
3. Parihar P, Singh S, Singh R, Singh VP, Prasad SM. Effect of salinity stress on plants and its tolerance strategies: a review. Environ. Sci. Pollut. Res. 2015;22:4056–4075.
4. Bellou S, Baeshen MN, Elazzazy AM, Aggeli D, Sayegh F, Aggelis G. Microalgal lipids biochemistry and biotechnological perspectives. Biotechnol. Adv. 2014;32:1476–1493.
